# Comparison of natural anticoagulant deficiency in cerebral venous thrombosis with deep venous thrombosis

**DOI:** 10.3892/mi.2025.218

**Published:** 2025-02-05

**Authors:** Song Giang Tran, Minh Phuong Vu, Thi Tuyet Mai Nguyen, Tuan Tung Nguyen, Phuong Thao Pham, Thi Hue Hoang, Hoang Vu, Thi Van Oanh Kieu, Hai Yen Duong

**Affiliations:** 1 Vietnam National Heart Institute, Bach Mai Hospital, Hanoi 11519, Vietnam; 2 Department of Hematology, Hanoi Medical University, Hanoi 11521, Vietnam; 3 Hematology and Blood Transfusion Center, Bach Mai Hospital, Hanoi 11519, Vietnam

**Keywords:** cerebral venous thrombosis, deep venous thrombosis, deficiency of natural anticoagulant, protein C, protein S, antithrombin III

## Abstract

The impact of a deficiency in natural anticoagulants on the occurrence of cerebral venous thrombosis (CVT) is controversial, as well as whether there is a difference between CVT and deep venous thrombosis (DVT). The present study aimed to evaluate the association between a deficiency in natural anticoagulants and the occurrence of CVT vs. DVT. For this purpose, 274 patients newly diagnosed with venous thromboembolism (VTE), including 114 patients with DVT (41.6%), 81 patients with CVT (29.6%) and 79 patients (28.8%) with another type of VTE were retrospectively analyzed. In addition, 219 patients without thrombosis were used as the control group. Protein C (PC), protein S (PS) and antithrombin III (AT III) assays were performed prior to commencing treatment. The rates of PC, PS, AT III deficiency in the VTE group were 23.7, 28.8 and 14.2%, respectively. The rates of PC, PS, AT III deficiency in the CVT group were 21, 29.6 and 7.4%, respectively. The rates of PC, PS, AT III deficiency in the DVT group were 28, 34.2 and 15.8%, respectively. There was no significant difference between the DVT and CVT groups. Univariable and multivariable regression analysis revealed that PS deficiency was associated with the occurrence of all VTE types, DVT and CVT with odds ratios of 1.895, 2.330 and 2.052, respectively. On the whole, the present study demonstrates that PS deficiency is associated with the occurrence of CVT. No marked differences were noted between the deficiency in natural anticoagulants and CVT and DVT. These results may prove to be useful in deciding whether to perform natural anticoagulants testing in patients with CVT.

## Introduction

Cerebral venous thrombosis (CVT) is an atypical presentation of venous thromboembolism (VTE), which is most commonly known in the form of deep venous thrombosis (DVT) or pulmonary embolism (PE) (1). However, CVT accounts for 0.5 to 3% of all stroke cases and can lead morbidity and mortality (2,3). Similar to VTE, the risk factors associated with CVT are Virchow's Triad, which includes hypercoagulability, blood stasis and vessel endothelial cell injury (4). Patients with inherited thrombophilia, such as a deficiency in natural anticoagulants [protein C (PC), protein S (PS) and antithrombin III (AT III)], factor V Leiden and prothrombin G20210A gene mutations or acquired thrombophilia as antiphospholipid syndrome (APS) have a predisposition for the development of VTE, including CVT (4,5). Homocysteinemia is either hereditary or acquired, and this has also been recognized to be associated with venous thrombosis (6,7). Hereditary thrombophilia is considered as a main cause of prothrombotic conditions. The mutation of factor V Leiden renders this factor resistant to activated PC. The prothrombin G20210A gene mutation can lead to higher levels of prothrombin. These two types of mutations both lead to the increased generation of thrombin. Activated PC inactivates factors Va and VIIIa. PS is a co-factor for activated PC in the inactivation of factors Va and VIIIa. AT III inhibits thrombin, factor Xa. All three of these anticoagulants play a role in reducing thrombin generation (5). These PC, PS and AT III deficiencies occur due to a loss of function, gene inactivation, or mutations in the *PROC*, *PROS1* and *SERPINC1* genes, respectively (8). A deficiency in natural anticoagulants can lead to an imbalance between the pro-coagulant and the anti-coagulant pathways. However, the impact of a deficiency in natural anticoagulants on the development of CVT remains controversial. Some researchers have suggested that factor V Leiden and prothrombin G20210A gene mutations, and APS are the most frequent causes, while deficiencies in natural anticoagulants, such as PC, PS and AT III are less common (4,9-11). However, other studies have demonstrated that a deficiency in natural anticoagulants is strongly associated with CVT, even more strongly than factor V Leiden and prothrombin G20210A gene mutations (12-15). Additionally, some researchers have investigated whether there is a difference in the deficiencies of natural anticoagulants between CVT and DVT (1,11,16).

The present study aimed to evaluate the association between a deficiency in natural anticoagulants and the occurrence of CVT vs. DVT.

## Patients and methods

### Patients

The present study was a retrospective cross-sectional study conducted at Bach Mai Hospital, Hanoi, Vietnam. From January, 2021 to December, 2021, all patients who were diagnosed with VTE and underwent PS, PC and AT III tests were consecutively recruited in the study. The inclusion criteria were all patients who were diagnosed with VTE and underwent PS, PC and AT III tests. The exclusion criteria were patients with previous thrombosis, or those treated with anticoagulants.

All patients who were subjected to natural anticoagulant tests were confirmed not to have thrombosis were enrolled as the control group (non-thrombosis group). Patients with previous thrombosis, or those treated with anticoagulants were excluded from the study.

The study protocol was approved by The Institutional Review Board of Bach Mai hospital (no. 5937/QĐ-BM). The patients provided verbal informed consent to participate in the study. All details of the patients were deidentified.

The patients with VTE were divided into three groups as follows: Group 1 included patients newly diagnosed with DVT, group 2 included patients newly diagnosed with CVT, and group 3 included patients with another type of VTE. The diagnosis of VTE, DVT and CVT was based on a Doppler ultrasound, computed tomography scan, or magnetic resonance imaging. Risk factors and comorbidities, such as being overweight, smoking, the use of contraceptives, immobility, post-operative status, infections, hypertension, dyslipidemia, diabetes, antiphospholipid syndrome and cancer were reported.

### Peripheral blood cell and coagulation tests

The analyses of white blood cells (WBCs), neutrophils, platelet count, PC, PS, AT III, fibrinogen and D dimer performed prior to commencing treatment.

The PC, PS, AT III assays was performed with ACL TOP 750 (Instrumentation Laboratory), using the HemosIL Protein C Kit, the HemosIL Free Protein S kit and the HemosIL Liquid Antithrombin kit, respectively. A PC activity between 70-140% was considered normal; a PS activity between 75-130% (for males) and 60-130% (for females) was considered normal; an AT III activity between 80-140% was also considered normal.

### Statistical analysis

Due to multiple comparisons being made, the differences in quantitative variables (age, hemoglobin, WBC, neutrophils, platelet count, fibrinogen and D dimer levels) were analyzed using one-way ANOVA or the Kruskal-Wallis test, depending on the variables of normal distribution or non-normal distribution (between two groups: VTE and non-thrombosis, DVT and non-thrombosis, CVT and non-thrombosis, CVT and DVT), followed by the Bonferroni correction (for parametric data) or Dunn's post hoc test (for non-parametric data). The Kolmogorov-Smirnov was used test to determine whether the data were normally or non-normally distributed. Differences in qualitative variables (sex, overweight, smoking, the use of contraceptives, immobility, post-operative status, infections, hypertension, dyslipidemia, diabetes, antiphospholipid syndrome, cancer, PC, PS and AT III deficiency) were analyzed using the χ^2^ or Fisher's exact tests (between two groups: VTE and non-thrombosis, DVT and non- thrombosis, CVT and non-thrombosis, CVT and DVT), followed by the Bonferroni correcdion. Binary logistic regression analysis was used to examine the association between the deficiency in anticoagulants (PC, PS and AT III) and the presence of types of thrombosis (VTE, DVT and CVT). SPSS 25 software (IBM Corp.) was used for the statistical analyses. A value of P<0.05 was considered to indicate a statistically significant difference.

## Results

### Patient characteristics

A total of 274 patients newly diagnosed with VTE, including 114 patients with DVT (41.6%), 81 patients with CVT (29.6%) and 79 patients (28.8%) with another type of VTE were enrolled in the present study (Table I). In addition, 219 patients without thrombosis were used as the control group. The patient data were retrospectively analyzed.

As demonstrated in Table II, no significant differences were found in factors, such as being overweight, smoking status, the use of contraceptives, immobility, post-operative status, infections, hypertension, dyslipidemia, diabetes, antiphospholipid syndrome and cancer between the VTE and non-thrombosis, DVT and non-thrombosis, CVT and non-thrombosis, and the CVT and DVT groups. However, the proportion of females in both the VTE and DVT group was higher than that in the non-thrombosis group (P=0.024 and 0.03, respectively). The age of the patients in the VTE and DVT groups was higher than that of the patients in the non-thrombosis group (P=0.036 and 0.006, respectively). Additionally, patients with CVT were likely to be younger than the patients with DVT (P=0.022).

As demonstrated in Table III, compared with the non-thrombosis group and CVT group, the patients in the DVT group had a lower hemoglobin level (P=0.004 and <0.001, respectively). The patients in the VTE group also had higher D dimer levels in comparison with the patients in the non-thrombosis group (P=0.033).

### Association between deficiencies in natural anticoagulants and the occurrence of types of VTE

The rates and frequencies of the deficiency in natural anticoagulants in the non-thrombosis, VTE, DVT and CVT groups are presented in Table III. The rates of PC, PS and AT III deficiency in the VTE group were 23.7, 28.8 and 14.2%, respectively. The rates of PC, PS and AT III deficiency in the DVT group were 28, 34.2 and 15.8%, respectively. The rates of PC, PS and AT III deficiency in the CVT group were 21, 29.6 and 7.4%, respectively. Generally, the rate and frequencies of PC, PS deficiency were high in the VTE, DVT and CVT groups. The VTE and DVT groups had higher rates of deficiencies in PC (P=0.034 and 0.009, respectively) and PS (P=0.003 and 0.001, respectively) than the group without thrombosis. Compared with the non-thrombosis group, the CVT group had a higher rate of PS deficiency (P=0.02). However, there was no significant difference between the DVT and CVT groups.

Univariable regression analysis revealed that all four factors, namely the female sex, age, and PC and PS deficiency were associated with the occurrence of VTE and DVT. However, following multivariable analysis, only the female sex and PS deficiency were associated with VTE, while the female sex, age and PS deficiency were associated with DVT (Tables IV and V). On the other hand, only PS deficiency was associated with CVT. Following analysis adjusted for sex and age, PS deficiency was indeed a factor associated with CVT (Table VI). Thus, univariable and multivariable regression analysis revealed that PS deficiency was associated with the occurrence of all types of VTE, DVT and CVT with odds ratios (ORs) of 1.895, 2.330 and 2.052, respectively (Tables IV, V and VI). Furthermore, the female sex was found to be a crucial factor associated with the occurrence of VTE and DVT with ORs of 1.535 and 1.654, respectively (Tables IV and V).

## Discussion

Hereditary thrombophilia, including a deficiency in natural anticoagulants, such as PC, PS and AT III has been recognized as a risk factor for the development of VTE, as well as CVT; however, the association, as well as the degree of influence on the occurrence of CVT remains to be determined. Previous studies have yielded differential results on the association between a deficiency in natural anticoagulants and the occurrence of CVT (4,9-15). This difference is mainly due to the different frequencies of a deficiency in natural anticoagulants in these studies.

The study by Kostal *et al* (10) demonstrated that in patients with CVT, the rates of deficiencies in PC, PS and AT III were 0, 1 and 0%, respectively. However, in the study by Bombeli *et al* (11), these three rates were 2%. Both of these studies were performed on European patients. Kumar *et al* (17) demonstrated that in Indian patients with CVT, the prevalence of PS deficiency was 7%; however, no cases were detected for PC or AT III deficiency. Wang *et al* (18) demonstrated that the prevalence rates of PC, PS and AT III deficiency in Chinese patients were 7.8, 5.2 and 0%, respectively. The present study demonstrated that in Vietnamese patients, the rate of PS deficiency was 29.6%, which was higher than that in the aforementioned studies, although markedly lower than that in the study by Daghriri *et al* (19) on Arabian patients (57%); the rate of AT III deficiency in the present study (7.4%) was also lower than that in the study by Daghriri *et al* (19) (13.7%). On the other hand, the rate of PC deficiency in the present study (21%) was higher than that (15.7%) in study by Daghriri *et al* (19). The deficiency in these natural anticoagulants appears to be dependent on race. Some studies have determined that a deficiency in natural anticoagulants is more common among Asian patients than in Caucasian patients. Additionally, Asians have been shown to be less likely to have factor V Leiden and prothrombin G20210A gene mutations in comparison to Caucasian patients (20-23). However, deficiencies in PC, PS and AT III are considered to be associated with *PROC, PROS1* and *SERPINC1* gene mutations, respectively (8,24-26), which may lead to differences in the rates of natural anticoagulant deficiencies between different races. Miyata *et al* (20) determined that the *PROS-K196E* mutation appears to be specific to Japanese patients, while Rojnuckarin *et al* (21) demonstrated that PROC mutations were common among Thai patients. Therefore, it is necessary to consider the association between the deficiency in natural anticoagulants and the occurrence of CVT according to race. However, the present study also demonstrated that a deficiency in PS was associated with the occurrence of CVT with an OR of 2.052 (95% CI, 1.127-3.737).

The present study did not identify any differences in the prevalence of a deficiency in natural anticoagulants between patients with CVT and DVT. However, Wysokinska *et al* (16) revealed the difference in inherited thrombophilia between CVT and DVT. The prothrombin G20210A gene mutation is common in CVT, whereas PC deficiency is more common in DVT (16). Bombeli *et al* (11) demonstrated that compared to patients with lower extremity deep thrombosis, patients with CVT had lower rates of deficiencies in PC, PS and AT III. However, Ordieres-Ortega *et al* (1) suggested that thrombophilia, including a deficiency in natural anticoagulants was more common in CVT than in classic VTE, such as DVT. In the present study, the degree of influence of a deficiency in PS on the occurrence of CVT and DVT was also approximately equivalent (OR, 2.052 and 2.330, respectively). Overall, further research is required to determine these differences.

The present study has certain limitations, which should be mentioned. The present study was a cross-sectional study. The patients were not followed-up to detect subsequent thrombotic events. Factor V Leiden and prothrombin G20210A gene mutations, and plasma homocysteine levels had were also not determined.

In conclusion, the present study demonstrates that a deficiency in PS is associated with the occurrence of all VTE, DVT and CVT with OR values of 1.895, 2.330 and 2.052, respectively. No differences were found in the deficiency of natural anticoagulants between patients with CVT and DVT. These results may be helpful in deciding whether to perform natural anticoagulant testing in patients with CVT.

## Figures and Tables

**Table I:** Location of VTE in the patients in the present study.

Location of thrombosis	No. of patients (%), n=274
Deep venous thrombosis	114 (41.6)
Cerebral venous thrombosis	81 (29.6)
VTE in another location	
Retina	4 (1.5)
Transverse sinuses	29 (10.6)
Spleen	7 (2.6)
Kidney	3 (1.1)
Mesentery	16 (5.8)
Heart	8 (2.9)
Pulmonary embolism	3 (1.1)

VTE, venous thromboembolism.

**Table II:** Risk factors and comorbidity of the patients.

Factor	Non-thrombosis (n=219)	VTE (n-274)	DVT (n=114)	CVT (n=81)	P-value
Age in years, median	41	45	47	41	**P1=0.036; P2=0.006;** P3>0.05; **P4=0.022**
Sex, n (%)					**P1=0.024; P2=0.03;** P3 and P4 >0.05 (χ^2^ test)
Male	131 (59.8)	136 (49.6)	54 (47.4)	40 (49.4)	
Female	88 (40.2)	138 (50.4)	60 (52.6)	41 (50.6)	
Overweight, n (%)					P1, P2 and P3 >0.05 (Fisher's exact test)
Yes	1 (0.5)	1 (0.4)	0 (0)	0 (0)	
No	218 (99.5)	273 (99.6)	114(100)	81(100)	
Smoking status, n (%)					P1>0.05 (χ^2^ test); P2, P3 and P4 >0.05 (Fisher's exact test)
Yes	9 (4.1)	12 (4.4)	5 (4.4)	3 (3.7)	
No	214 (95.9)	262 (95.6)	109 (95.6)	78 (96.3)	
Contraception, n (%)					P1>0.05 (χ^2^ test); P2, P3 and P4 >0.05 (Fisher's exact test)
Yes	7 (3.1)	7 (2.6)	4 (3.5)	1 (1.2)	
No	212 (96.9)	217 (97.4)	110 (96.5)	80 (98.8)	
Immobility, n (%)					P1>0.05 (χ^2^ test); P2, P3 and P4 >0.05 (Fisher's exact test)
Yes	8 (3.7)	5 (1.8)	2 (0.9)	1 (1.2)	
No	211 (96.7)	269 (98.2)	112 (99.1)	80 (98.8)	
Post-surgery, n (%)					P1, P2, P3 and P4>0.05 (χ^2^ test)
Yes	23 (10.5)	26 (9.5)	11 (5.1)	10 (12.3)	
No	196 (89.5)	248 (90.5)	103 (94.9)	71 (87.7)	
Infection, n (%)					P1, P2, P3 and P4 >0.05 (χ^2^ test)
Yes	44 (20.1)	41 (15.0)	19 (16.7)	14 (17.3)	
No	175 (79.9)	233 (85.0)	95 (83.3)	67 (82.7)	
Hypertension, n (%)					P1, P2, P3 and P4 >0.05 (χ^2^ test)
Yes	53 (24.2)	66 (24.1)	26 (22.8)	21 (25.9)	
No	166 (75.8)	108 (75.9)	88 (77.2)	60 (74.1)	
Dyslipidemia, n (%)					P1 >0.05 (χ2 test); P2, P3 and P4 >0.05 (Fisher's exact test)
Yes	8 (3.7)	10 (3.6)	3 (2.6)	1 (1.2)	
No	211 (96.3)	264 (96.4)	111 (97.4)	80 (98.8)	
Diabetes, n (%)					P1, P2, P3 and P4 >0.05 (χ^2^ test)
Yes	19 (8.7)	28 (10.2)	11 (9.6)	10 (12.3)	
No	200 (91.3)	246 (89.8)	103 (90.4)	71 (87.7)	
Anti-phospholipid syndrome, n (%)					P1, P2, P3 and P4 >0.05 (χ^2^ test)
Yes	19 (8.7)	20 (7.3)	7 (6.1)	9 (1.1)	
No	200 (91.3)	254 (92.7)	107 (93.9)	72 (98.9)	
Cancer, n (%)					P1, P2 >0.05 (χ^2^ test)' P3 and P4 >0.05 (Fisher's exact test)
Yes	11 (5.0)	14 (5.1)	9 (7.9)	2 (2.4)	
No	208 (95.0)	260 (94.9)	105 (92.1)	79 (97.6)	

Values in bold font indicate statistically significant differences (P<0.05). P1, P-value for comparison between the VTE and non-thrombosis group; P2, P-value for comparison between the DVT and non-thrombosis group; P3, P-value for comparison between the CVT and non-thrombosis group; P4, P-value for comparison between the DVT and CVT groups; VTE, venous thromboembolism; DVT, deep venous thrombosis; CVT, cerebral venous thrombosis.

**Table III:** Laboratory indices of the patients.

Indices	Non-thrombosis (n=219)	VTE (n-274)	DVT (n=114)	CVT (n=81)	P-value
Hemoglobin (g/l), mean ± SD	132.14±25.84	129.53± 24.50	123.41± 24.81	137.03± 22.24	P1 >0.05; **P2=0.004;** P3 >0.05; **P4 <0.001**
WBC (g/l), median	10.48	10.40	9.70	10.80	P1, P2, P3 and P4 >0.05
Neutrophils (g/l), median	7.7	7.33	6.66	7.70	P1, P2, P3 and P4 >0.05
Platelets (g/l), median	249	247	241	257	P1, P2, P3 and P4 >0.05
Fibrinogen (g/l), mean ± SD	3.76±1.50	4.00±1.40	3.79±1.36	3.88±1.25	P1, P2, P3 and P4 >0.05
D dimer (mg/l FEU), median	2.00	3.6	4.66	2.95	**P1=0.033;** P2=0.05; P3 and P4 >0.05
PC decrease, n (%)					**P1=0.034; P2=0.009;** P3 and P4 >0.05 (χ^2^ test)
Yes	35(16)	65 (23.7)	32 (28.0)	17(21)	
No	184(84)	209 (76.3)	82(72)	64(79)	
PS decrease, n (%)					**P1=0.003; P2=0.001; P3=0.02;** P4 >0.05 (χ^2^ test)
Yes	38 (17.4)	79 (28.8)	39 (34.2)	24 (29.6)	
No	181 (82.6)	195 (71.1)	75 (65.8)	57 (70.4)	
AT III decrease, n (%)					P1, P2, P3 and P4 >0.05 (χ^2^ test)
Yes	34 (15.5)	39 (14.2)	18 (15.8)	6 (7.4)	
No	185 (84.5)	235 (85.8)	96 (84.2)	75 (92.6)	

Values in bold font indicate statistically significant differences (P<0.05). P1, P-value for comparison between the VTE and non-thrombosis group; P2, P-value for comparison between the DVT and non-thrombosis group; P3, P-value for comparison between the CVT and non-thrombosis group; P4, P-value for comparison between the DVT and CVT groups; VTE, venous thromboembolism; DVT, deep venous thrombosis; CVT, cerebral venous thrombosis; PS, protein S; PC, protein C; AT III, anti-thrombin III.

**Table IV:** Univariable and multivariable logistic regression analysis for a deficiency in natural anticoagulants associated with the presence of VTE.

A, Univariable logistic regression						
Factor	B	S.E	Wald	P-value	OR	95% CI
Age	0.012	0.006	4.160	**0.041**	1.012	1.000-1.024
Female sex	0.412	0.183	5.064	**0.024**	1.511	1.055-2.163
PC decrease	0.492	0.233	4.462	**0.035**	1.635	1.036-2.580
PS decrease	0.657	0.223	8.707	**0.003**	1.930	1.247-2.986
B, Multivariable logistic regression						
Factor	B	S.E	Wald	P-value	OR	95% CI
Age	0.010	0.006	2.877	0.090	1.010	0.998-1.022
Female sex	0.429	0.187	5.261	**0.022**	1.535	1.064-2.214
PC decrease	0.423	0.239	3.147	0.076	1.527	0.957-2.437
PS decrease	0.639	0.226	8.012	**0.005**	1.895	1.217-2.951

Values in bold font indicate statistically significant differences (P<0.05). VTE, venous thromboembolism; PC, protein C; PS, protein S; OR, odds ratio; CI, confidence interval; S.E, standard error.

**Table V:** Univariable and multivariable logistic regression analysis for a deficiency in natural anticoagulants associated with the presence of DVT.

A, Univariable logistic regression						
Factor	B	S.E	Wald	P-value	OR	95% CI
Age	0.020	0.007	7.775	**0.005**	1.020	1.006-1.034
Female sex	0.503	0.233	4.674	**0.031**	1.654	1.048-2.610
PC decrease	0.719	0.278	6.667	**0.010**	2.052	1.189-3.540
PS decrease	0.907	0.266	11.616	**0.001**	2.477	1.470-4.173
B, Multivariable logistic regression						
Factor	B	S.E	Wald	P-value	OR	95% CI
Age	0.017	0.007	5.450	**0.020**	1.017	1.003-1.032
Female sex	0.503	0.241	4.342	**0.037**	1.654	1.030-2.655
PC decrease	0.540	0.292	3.415	0.065	1.716	0.968-3.043
PS decrease	0.846	0.275	9.478	**0.002**	2.330	1.360-3.992

Values in bold font indicate statistically significant differences (P<0.05). DVT, deep venous thrombosis; PC, protein C; PS, protein S; OR, odds ratio; CI, confidence interval; S.E, standard error.

**Table VI:** Logistic regression analysis for a deficiency in natural anticoagulants associated with the presence of CVT.

A, Univariable logistic regression						
Factor	B	S.E	Wald	P-value	OR	95% CI
PS decrease	0.696	0.302	5.319	**0.021**	2.006	1.110-3.623
B, Adjusted with age and sex						
Factor	B	S.E	Wald	P-value	OR	95% CI
PS decrease	0.719	0.306	5.524	**0.019**	2.052	1.127-3.737

Values in bold font indicate statistically significant differences (P<0.05). CVT, cerebral venous thrombosis; PS, protein S; OR, odds ratio; CI, confidence interval; S.E, standard error.

## Data Availability

The data generated in the present study may be requested from the corresponding author.
